# Nutritional guidance in spondyloarthritis: confronting the evidence gap

**DOI:** 10.1097/BOR.0000000000001090

**Published:** 2025-03-17

**Authors:** Roberta Ramonda, Giacomo Cozzi, Francesca Oliviero

**Affiliations:** Rheumatology Unit, Department of Medicine - DIMED, University of Padova, Padova, Italy

**Keywords:** dietary intervention, Mediterranean diet, nutritional guidance, spondyloarthritis

## Abstract

**Purpose of review:**

to summarize current evidence on the role of specific dietary patterns in spondyloarthritis (SpA) management.

**Recent findings:**

dietary interventions may offer a novel, complementary strategy to manage symptoms and enhance overall quality of life in many rheumatic diseases, including SpA. Evidence suggests that the Mediterranean diet may have beneficial effects on inflammation and SpA symptoms. Although there is growing interest in the ketogenic diet with some promising results, data is scarce. Some SpA patients may have sensitivities or intolerances to certain foods containing gluten, which can trigger or worsen their symptoms, especially when associated with intestinal inflammation. Hypocaloric diets and weight loss can provide significant benefit in overweight and obese patients with SpA, potentially reducing systemic inflammation. Finally, while the efficacy of probiotics remains a matter of debate, periods of fasting have proven effective in reducing disease activity indices.

**Summary:**

the importance of a healthy dietary lifestyle and its potential benefits in symptom management is acknowledged by the majority of the patients. There is an increased need and demand from patients to receive nutritional counseling that should be integrated into routine SpA management to enhance patient outcomes.

## INTRODUCTION

Spondyloarthritis (SpA) are a family of recurrent chronic diseases characterized by a wide spectrum of articular and extra-articular manifestations. It is classified into axial SpA (axSpA), involving the spine and sacroiliac joints, and peripheral SpA, which includes psoriatic arthritis (PsA).

While the pathogenesis of SpA is not yet fully understood, it involves a complex interplay between genetic predisposition – such as the presence of HLA-B27 – and environmental factors, including gut dysbiosis. The role of intestinal microbiota alterations in the pathogenesis of immune-mediated diseases, including inflammatory bowel disease (IBD) and SpA, has gained increasing attention [1^▪^,^2^]. Environmental factors that disrupt the gut microbiome, such as dietary habits and antibiotic use, may contribute to disease onset and progression [3]. 

**Box 1:**
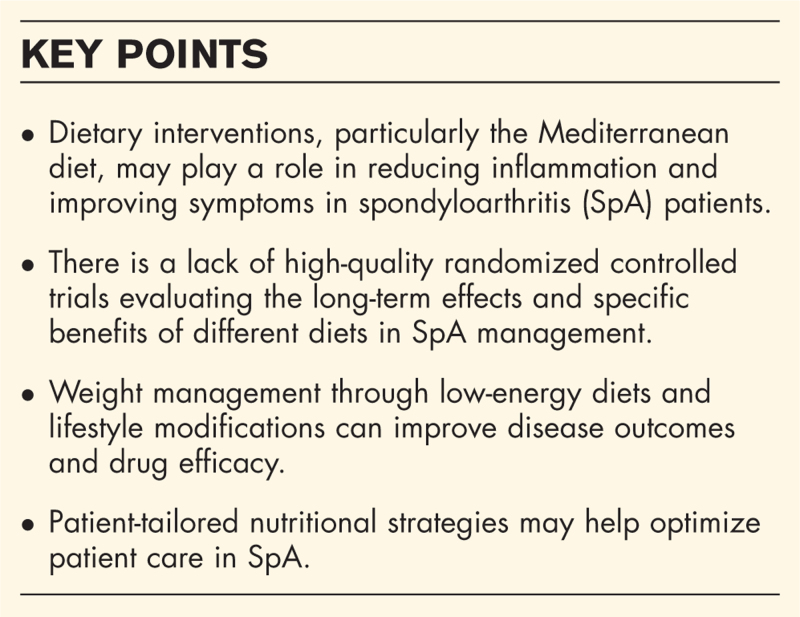
no caption available

Western dietary patterns, characterized by high intake of saturated fats and refined carbohydrates, have been linked to an increased prevalence of metabolic and cardiovascular comorbidities, as well as a pro-inflammatory state that may exacerbate autoimmune diseases [4]. Although various pharmacological treatments exist for SpA, dietary interventions have emerged as a complementary strategy to manage symptoms and enhance overall well being. Evidence suggests that certain dietary patterns, such as the Mediterranean diet, rich in fruits, vegetables, whole grains, and omega-3 fatty acids, may have beneficial effects on SpA symptoms and inflammation [5^▪^]. Conversely, processed foods, saturated fats, and refined sugars may exacerbate inflammation and worsen SpA symptoms. Additionally, some individuals with SpA may experience sensitivities or intolerances to certain foods containing gluten or dairy, which can trigger or worsen their symptoms.

Different dietary approaches have been proposed for SpA patients, each with distinct characteristics and potential benefits (Table 1). This review aims to highlight currently available evidence on the effectiveness of specific dietary patterns as a modifiable factor in the therapeutic approach in patients with SpA.

**Table 1:** Types of diets that have been considered in patients with SpA

Dietary pattern	Characteristics	Who might benefit
MD	Whole grains, fruits, vegetables, seafood, beans, nuts, healthy fats (EVO oil)	All patients
KD	Low carbohydrate intake, moderate amount of protein, high intake of fats	Patients with normal lipid profile
GFD	Elimination of foods containing gluten (wheat, barley, rye)	Patients with celiac disease or gluten sensitivity
LED	Calories restriction with all essential nutritional requirements	Overweight/obese patients
PBD	Foods primarily from plants	All patients
FAS	Period or intermittent fasting	Disease under control, not pregnant, no diabetes, no adolescents, no eating disorders

FAS, fasting; GFD, gluten-free diet; KD, ketogenic diet; LED, low-energy diet; MD, Mediterranean diet; PBD, plant-based diet; SpA, spondyloarthritis.

## MEDITERRANEAN DIET

The Mediterranean diet (MD) is globally promoted for its health benefits. Rich in antioxidants, omega-3 fatty acids, polyphenols, and healthy fats (e.g., olive oil), while low in processed foods, red meat, and unhealthy fats, the MD is considered an anti-inflammatory diet [6]. The beneficial effects of the MD cannot be attributed to individual components alone but rather to the synergistic combination of various macro- and micronutrients.

Several observational studies have evaluated the MD as a potential adjunct to conventional therapy for ax-SpA and PsA, revealing that lower adherence to the MD is associated with higher disease activity [5^▪^,^7^]. More recently, a study on 355 patients with PsA and psoriasis (PsO) investigated the impact of MD adherence and physical exercise on disease outcomes. Although higher adherence to the MD was associated with reduced inflammatory indices and skin disease severity, only exercise showed a significant correlation with disease activity in PsA; diet demonstrated a significant association only with enthesitis [8]. Another recent observational study examined the impact of dietary habits on inflammatory arthritis (rheumatoid arthritis and SpA), analyzing dietary information collected via a certified app from 744 patients. The study assessed major dietary patterns, including MD, vegetarian/vegan, and low-carbohydrate diets, as well as the consumption of specific food groups such as processed meats, fatty fish, fruits, and sugars. Patients adhering to the MD or consuming higher amounts of fatty fish (omega-3) experienced a significant reduction in pain compared to those not following a specific diet. However, this difference was not significant for those adhering to a vegetarian/vegan or low-carbohydrate diet [9].

Although observational studies suggest potential benefits, randomized controlled trials (RCTs) are still needed to establish more definitive recommendations regarding the role of MD in PsA management.

Patients with SpA often present with multiple cardiovascular and metabolic comorbidities. In this context, beyond its anti-inflammatory benefits, the MD has been shown to promote weight loss in overweight and obese individuals, reduce cardiovascular disease risk, and improve overall mortality. Meta-analyses of randomized trials have demonstrated that the MD leads to a significant reduction in body weight [10,11]. The PREDIMED trial, a landmark study on the cardiovascular effects of the MD, randomized 7447 participants into three dietary intervention groups. Participants following an MD enriched with olive oil or nuts showed a lower incidence of major cardiovascular events, including myocardial infarction and stroke, compared to those following a low-fat diet [12].

## KETOGENIC DIET

In recent years, the ketogenic diet (KD) has been increasingly considered as an alternative nutritional strategy for many diseases, such as obesity, type 2 diabetes, cardiovascular and neurological diseases. This dietary approach is characterized by a drastic reduction in carbohydrate intake, a moderate amount of protein, and a high proportion of fats, leading to a metabolic state known as ketosis. The consequent production of ketone bodies and free fatty acids, including beta-hydroxybutyrate (BHB), has been linked to its anti-inflammatory effects. Among these, some molecular events that have been demonstrated in experimental models include the inhibition of NLRP3 inflammasome and the reduction of oxidative stress through the improvement of mitochondrial function [13,14^▪^]. This pathophysiological background explains the growing interest in the effect of KD in inflammatory diseases.

Evidence on SpA is limited to a recently published study investigating the effects of KD as compared to MD on clinical and biochemical markers of inflammation in obese patients with psoriasis and PsA [15^▪▪^]. In that study, the patients were randomly assigned to either the KD or MD group for 8 weeks and, after a washout period, they crossed over to the other diet for another 8 weeks. Compared to MD, the KD led to significant improvements in both clinical markers of disease activity, including the Psoriasis Area and Severity Index (PASI) and the Disease Activity Index of Psoriatic Arthritis (DAPSA), and inflammatory markers, such as interleukin (IL)-6, IL-17, and IL-23.

The effects of the KD were previously observed in PsO wherein this dietary intervention appeared to have a role in correcting the aminoacidic dysmetabolism typically observed in these patients. In this regard, a metabolomics analysis conducted after a 4-week KD revealed a correction of the dysmetabolic pathways, including an increase in hydroxybutyrate and a decrease in pyruvic acid, choline, leucine and alanine levels [16]. A decrease in the levels of certain cytokines was also described, thus supporting the anti-inflammatory properties of this dietary pattern.

Notwithstanding these promising effects on dysmetabolism, inflammation, and clinical symptoms, further research is needed to confirm these findings and determine the long-term effects of the KD in SpA, including potential risks and side effects.

## GLUTEN-FREE DIET

The popularity of the gluten-free diet has increased rapidly in recent years, especially among patients with inflammatory conditions, such as rheumatological and inflammatory bowel diseases (IBD). These patients perceive gluten-free eating as a healthier lifestyle choice even in absence of celiac disease or gluten intolerance [17].

Regarding SpA, an increased prevalence of raised IgA antibodies to gliadin and of celiac disease has been described in the past among patients with PsA but data has not been further examined [18]. Similarly, an increased sensitivity to gluten has been shown in patients with SpA, even in the absence of celiac disease [19].

Data from large prospective epidemiological studies, including the Nurses’ Health Study I, II, and the Health Professionals Follow-up Study, found no association between gluten intake and the risk of developing IBD, PsO, PsA and atopic dermatitis in a cohort of 208 280 US subjects [20]. The gluten-free dietary approach has gained some attention in patients with SpA for its potential effects in subclinical intestinal inflammation which is often present in these patients along with conditions like Crohn's disease and irritable bowel syndrome. As gluten has been implicated in intestinal inflammation and increased intestinal permeability in some individuals, eliminating gluten is thought to reduce intestinal inflammation and consequently systemic inflammation in SpA [21].

The dysbiosis observed in patients with SpA and the similarity in the microbiota of patients with SpA with those with celiac disease led some authors to design a protocol for a randomized, double-blind, placebo-controlled, multicenter trial to investigate the impact of a gluten-free diet on the quality of life in patients with SpA which is still recruiting patients [22].

A recent multicenter prospective study conducted in a cohort of 193 participants with a chronic inflammatory disease diagnosis (i.e., Crohn's disease, ulcerative colitis, rheumatoid arthritis, axSpA, PsA or PsO) demonstrated that gluten intake has no impact on response to biological treatment [23^▪▪^]. However, the same study showed that patients with a high gluten intake reported lower health-related quality of life than those with a low-to-moderate gluten intake.

The scientific evidence for the efficacy of gluten-free diets in managing nonceliac inflammatory diseases such as SpA remains limited and inconclusive.

## LOW ENERGY DIET AND WEIGHT LOSS

The rationale for using a low energy diet in patients with SpA is based on factors mainly linked to obesity. SpA, particularly PsA, are often associated with an increased risk of obesity [24]. The chronic low-grade inflammatory state that characterized obesity through the increase of cytokines, chemokines and adipokines, can exacerbate systemic inflammation and disease activity in the patients. It has been observed that PsA patients with obesity have higher DAPSA as compared to nonobese patients and those with higher bone mass index (BMI) are less likely to achieve sustained minimal disease activity state compared to those with lower BMI [25,26].

A significant aspect of the low energy diet is the improved response to medications. It has been observed that obesity is associated with higher odds antitumor necrosis factor (TNF) treatment failure as compared to nonobese patients [27^▪^]. Some studies have suggested that weight loss induced by a low energy diet may improve the response to therapy, such as anti-TNF drugs [28^▪^]. Furthermore, weight loss can reduce the mechanical load on the joints, alleviating pain and improving joint mobility and quality of life in patients with SpA. Finally, the results from the DIETA randomized clinical trial support the use of a hypocaloric diet independently of weight loss. A 12-week hypocaloric dietary intervention improved joint disease activity in PsA patients, regardless of weight loss; and the likelihood of achieving disease remission was linked to overall diet quality. Adding omega-3 supplementation was more effective than a hypocaloric diet alone in promoting weight loss, beneficial body composition changes, including fat mass and waist circumference reduction, but had no extra beneficial effects on disease activity [29].

While more research is still needed, current evidence suggests that weight loss through caloric restriction can provide significant benefits in overweight and obese patients with SpA potentially reducing systemic inflammation, improving quality of life, and increasing the efficacy of medical treatments.

## GUT MICROBIOTA, PROBIOTICS, AND PREBIOTICS

A growing body of evidence shows that alterations in the gut microbiome composition may play a crucial role in the development and progression of SpA. The gut microbiome is a heterogeneous community of microorganisms that plays a crucial role in maintaining intestinal homeostasis. The gut microbiota appears to be fundamental in immune system modulation from early life; in fact, breastfeeding constitutes one of the richest sources of microbial colonization in neonates [30]. A recent single-center, retrospective, case-control study including 195 children with juvenile SpA and matched controls suggested that shorter exclusive breastfeeding duration or its absence may be associated with an increased risk of developing juvenile SpA [31^▪▪^].

Emerging evidence indicates that gut dysbiosis may precede disease onset, leading to increased intestinal permeability and subsequent immune system activation via IL-17/IL-23 inflammatory pathways through mechanisms such as molecular mimicry, altered apoptosis, and modulation of inflammatory responses [32]. Recent Mendelian randomization studies have provided insights into how gut dysbiosis may influence PsA development. One study assessed the association between gut microbiota and PsO using genome-wide association study (GWAS) data from 337 159 patients and 433 201 controls, identifying eight bacterial taxa significantly associated with the disease, some correlated with increased risk and others with potential protective effects [33]. Another Mendelian randomization study investigated the genetic link between gut microbiota and PsA risk using GWAS data from 2776 PsA patients and 221 323 controls. The analysis identified three bacterial genera (Blautia, Eubacterium fissicatena group, Methanobrevibacter) associated with an increased PsA risk and one genus (Ruminococcaceae UCG-002) with a protective effect. No reverse causality was observed, suggesting that PsA may not significantly alter microbiota composition, but rather that dysbiosis may act as a disease trigger [34].

Thanks to its high fiber and polyphenol contents, MD has been associated with gut microbiota improvements, including an increase in beneficial bacteria such as *Faecalibacterium prausnitzii* and *Bifidobacterium* spp., along with a reduction in IL-17 levels [35].

Exploring the use of probiotics and prebiotics to modulate gut microbiota and reduce disease activity in SpA may offer new therapeutic perspectives. Probiotics are live microorganisms contained in foods or supplements that improve intestinal microbial homeostasis, whereas prebiotics are nondigestible fibers that nourish beneficial gut bacteria, enhancing nutrient absorption and microbiota composition [36]. An open-label pilot study in 10 PsA patients demonstrated reduced disease activity and intestinal permeability (which is altered in PsA) following 12 weeks of oral probiotic administration [37]. However, these effects were not long-lasting. Conversely, a retrospective observational study of 782 PsA patients found no significant differences in clinical outcomes between patients who used probiotics and those who did not [38].

While the efficacy of probiotics in PsA management remains to be fully elucidated, more robust evidence exists regarding PsO. A double-blind, randomized clinical trial on 50 patients with plaque PsO showed that an 8-week supplementation with a probiotic-containing beverage led to significant improvements in inflammatory indices, the Dermatology Life Quality Index (DLQI), and PASI [39]. A systematic review and meta-analysis of three RCTs including 164 PsO patients confirmed that probiotics can improve PASI scores, reduce C-reactive protein (CRP) and TNF levels, without significant adverse effects [39]. A 12-week single-center clinical trial involving 63 PsO patients, approximately half of whom had PsA, assessed the response to Bacillus-based probiotics and prebiotics. The results indicated a reduction in PASI and DLQI scores, BMI, and inflammatory cytokines TNF-α, IL-6, Interferon (IFN)-γ, along with an improvement in gut microbiota diversity [40].

## OTHER INTERVENTIONS AND UNMET NEEDS

There is scant evidence on the role of other types of diet such as fasting, vegetarian/vegan, or elimination diet in SpA. A predominantly plant-based diet rich in fruits, vegetables, whole grains, and legumes may provide antioxidants and anti-inflammatory bioactive compounds that may help in managing symptoms in arthritis [41]. Periods of fasting have been shown to induce a shift in the composition and diversity of gut microbiome favoring the proliferation of certain bacterial species that may confer health benefits. Intermittent Ramadan fasting has proven effective in reducing Bath Ankylosing Spondylitis Disease Activity Index (BASDAI), PASI and DAPSA scores in a cohort of 37 patients with PsA, highlighting the need for further research and clinical trials to establish the role of fasting in chronic inflammatory diseases [42].

Nevertheless, some dietary patterns appear to be unhealthy for patients. A diet characterized by high intake of animal proteins and sodium, as well as low fiber intake and plant-based foods may negatively influence systemic inflammation and disease activity. This type of diets present a high dietary acid load (DAL). A recent study highlighted the potentially detrimental relationship between increased DAL and disease severity in patients with PsA. Despite the small sample size, a significant association was found between DAL and higher disease activity scores (DAPSA and DAS28), as well as increased inflammatory markers (CRP) [43].

Ultra-processed foods have also gained increasing attention for their harmful impact on health. These energy-dense, nutrient-poor, easy to eat foods typically contain little or no whole foods and are high in fat, sugar, and/or salt and additives. Although no data are currently available regarding their potential implications in SpA, these foods have low nutritional values and have been consistently associated with obesity and chronic diseases [44^▪▪^,^45^,46^▪^].

Dietary supplementation aims to correct nutritional deficiencies using concentrated formulations, such as pills, powders, or liquids. Vitamin D is one of the most widely prescribed, for its well-known crucial role in promoting bone health, boosting immune function, and regulating inflammatory processes. Two systematic reviews have confirmed that patients with PsA and axSpA tend to have lower circulating vitamin D levels [47,48]. Vitamin D supplementation has been suggested as an adjuvant to reduce disease activity and improve clinical outcomes, particularly in patients with deficient baseline levels. Mendelian randomization studies have identified a causal link between serum calcifediol levels and PsO, consistent with the therapeutic use of vitamin D analogues in the management of PsO.

Lifestyle counseling, including dietary recommendations, can have a positive impact in the management of inflammatory joint diseases. However, in routine rheumatology practice, these interventions are not systematically implemented. Studies have reported that only a minority of patients receive nutritional counseling from clinicians. A recent multicenter cross-sectional study found that only 18% of axSpA patients received dietary advice directly from their treating physician [49^▪^]. In this context, individualized lifestyle counselling provided by a mobile application could overcome some common obstacles encountered by patients. A very recent study demonstrated that patients receiving lifestyle guidance through such an application had a higher likelihood of achieving low disease activity or remission compared to a control group. Additionally, the adherence to the MD improved, suggesting a positive effect of the application on dietary awareness and habits [50^▪▪^].

## NUTRITIONAL GUIDANCE

Although dietary advice should be tailored to each patient according to their symptoms, comorbidities and nutritional preferences, some general guidelines can be taken into consideration to maintain healthy dietary habits in patients with SpA (Table 2).

**Table 2:** Some nutritional guidance for patients with SpA

• Adopt a healthy and balanced diet such as the Mediterranean diet • Consume anti-inflammatory foods, including green leafy vegetables, fruits, nuts, omega 3-rich fish • Consider probiotics and prebiotics to support gut health, especially in presence of SpA-related gut dysbiosis. • Avoid refined carbohydrates, fried foods, sugar-sweetened beverages, processed meat, fatty condiments which may contribute to systemic inflammation. • Maintain a healthy weight as overweight and obesity exacerbates SpA symptoms and reduces medication efficacy. • Use specific dietary approaches (e.g., keto diet, gluten-free diet) only after consultation with a nutritionist or a healthcare professional

SpA, spondyloarthritis

## CONCLUSION

Despite increasing scientific findings supporting the role of dietary interventions in SpA, several knowledge gaps remain (Table 3). Current evidence suggests that dietary interventions, particularly adherence to the MD and weight management strategies, may play a supportive role in managing spondyloarthritis. However, robust randomized controlled trials are needed to establish causality and optimize dietary recommendations. Future research should focus on defining the role of gut microbiota in the pathogenesis of SpA, and identifying patient-tailored nutritional strategies that account for disease heterogeneity and comorbidities. Nutritional counseling should be integrated into routine SpA management to enhance patient outcomes.

**Table 3:** Unmet needs in nutritional research in SpA

Unmet needs	Future research implementation
High-quality RCTs	Conduct randomized controlled trials on different dietary patterns to assess impact on disease indices
Role of gut microbiota	Metabolomics and microbiome studies to correlate diet with microbiota composition
Personalization of dietary recommendations	Precision medicine approaches based on individual characteristics (e.g., genetics, microbiota, inflammation)
Diet-drug interactions	Analyses on how diets may influence the effectiveness of biologic and symptomatic therapies
Nutritional education in clinical care	Greater integration of nutritionists into rheumatology teams to provide personalized dietary guidance

SpA, spondyloarthritis.

## Acknowledgements

*The authors wish to thank Eric Franck Nde for editing the English version.*

### Financial support and sponsorship

*None.*

### Conflicts of interest

*There are no conflicts of interest.*

## REFERENCES AND RECOMMENDED READING

Papers of particular interest, published within the annual period of review, have been highlighted as:

▪ of special interest
▪▪ of outstanding interest
